# How can risk of COVID-19 transmission be minimised in domiciliary care for older people: development, parameterisation and initial results of a simple mathematical model

**DOI:** 10.1017/S0950268821002727

**Published:** 2021-12-17

**Authors:** István Z. Kiss, Konstantin B. Blyuss, Yuliya N. Kyrychko, Jo Middleton, Daniel Roland, Lavinia Bertini, Leanne Bogen-Johnston, Wendy Wood, Rebecca Sharp, Julien Forder, Jackie A. Cassell

**Affiliations:** 1 Department of Mathematics, University of Sussex, Falmer, Brighton BN1 9QH, UK; 2 Department of Primary Care and Public Health, Brighton and Sussex Medical School, Brighton BN1 9PH, UK; 3 PSSRU, School for Social Policy, Sociology and Social Research, University of Kent, Canterbury, Kent, UK; 4 School of Psychology, University of Sussex, Brighton, UK; 5 School of Health Sciences, University of Brighton, Brighton, UK; 6 Kent Surrey Sussex Academic Health Science Network, Worthing, West Sussex, UK

**Keywords:** care workers, domiciliary care, networks, SEIR/D

## Abstract

This paper proposes and analyses a stochastic model for the spread of an infectious disease transmitted between clients and care workers in the UK domiciliary (home) care setting. Interactions between clients and care workers are modelled using specially generated networks, with network parameters reflecting realistic patterns of care needs and visit allocation. These networks are then used to simulate a susceptible-exposed-infected-recovered/dead (SEIR/D)-type epidemic dynamics with different numbers of infectious and recovery stages. The results indicate that with the same overall capacity provided by care workers, the minimum peak proportion of infection and the smallest overall size of infection are achieved for the highest proportion of overlap between visit allocation, i.e. when care workers have the highest chances of being allocated a visit to the same client they have visited before. An intuitive explanation of this is that while providing the required care coverage, maximising overlap in visit allocation reduces the possibility of an infectious care worker inadvertently spreading the infection to other clients. The model is generic and can be adapted to any directly transmitted infectious disease, such as, more recently, corona virus disease 2019, provided accurate estimates of disease parameters can be obtained from real data.

## Introduction

The catastrophic impact of corona virus disease 2019 (COVID-19) in care homes for older people has been well documented [1, 2], demonstrating the extreme vulnerability of their frail elderly residents receiving personal care to this emerging infection. Less well recognised is the large number of similarly frail older people receiving professional (‘regulated’) care in their own homes by visiting carers who visit many clients in different households, known as home care or domiciliary care. In 2020, an estimated 715 000 people provided care to around 330 000 community care users above 65 years old in England [3, 4]. Vacancy in the domiciliary-care-providing sector is typically 1 in 10, with annual staff turnover of 1 in 5 [3]. Social care, both residential and domiciliary, can contribute to transmission of COVID-19 and other infections such as influenza or norovirus (winter vomiting). Providers may become unable to deliver care if staff are ill or isolating, putting vulnerable people at risk.

Although outbreaks in care homes are quickly recognised and can have devastating effects, they are harder to detect in domiciliary care, and data are poor. Although there was a rise in notifications of death to the Care Quality Commission in England, these relate only to deaths during the actual delivery of or as a result of care [5]. A prevalence study of domiciliary care workers was comparable with the wider population, but under sampled individuals off work due to COVID-19 and did not include antibody tests [6]. Dispersed across households and protected from knowledge of each other by the confidentiality required of carers and agencies, domiciliary care associated outbreaks of infection can be hard to detect and are probably under-ascertained. The few available studies in domiciliary care demonstrate this difficulty in relation to scabies [7] and more recently COVID-19 [8].

Research on domiciliary care remains scarce [9], and particularly its role in transmission and prevention of infection, although this work connects client households through care workers, and also creates connections with carers' own households. Around one-fifth of domiciliary care agencies reported providing care to at least one person with suspected or confirmed COVID-19 [10]. Given the vulnerability of domiciliary care clients to severe outcomes from COVID-19 and other infections, and the need to protect visiting carers and their families from exposure, there is a need to understand transmission dynamics in this neglected and difficult to study setting.

Since the start of COVID-19 pandemic, a large number of mathematical models have looked into disease dynamics from the perspective of evaluating the effectiveness of different non-pharmaceutical interventions [11–15], and assessing various scenarios of lifting lockdown restrictions [16, 17]. Some research has been conducted on modelling COVID-19 transmission in the care homes [18], where this disease has been associated with a significant death toll, but so far it has not been studied in the domiciliary care setting. We aim to fill this learning gap by developing and parameterising a simple mathematical model of transmission through domiciliary care and use it to evaluate the potential impact of different policies for the allocation of carers to clients.

## Mathematical model

**Fig. 1.**
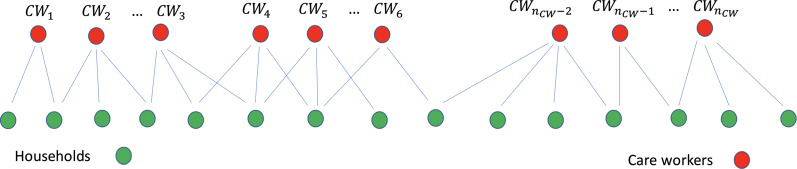
Pictorial representation of the care worker and client/household interactions. Some of these links will be weighted depending on the number of repeat or return visits of care workers to the same client.

We model interactions between clients (people receiving care within their own household) and care workers using a bi-partite network, as shown in Figure 1. The infection can be passed from care workers to clients, and from clients to care workers. Most of the transmission will be associated with the movement of care workers between clients in different households.

The aim of this study is to understand the impact of care workers' working pattern on the spread of infectious diseases. We are interested in the difference between whether clients are visited by the same or different care workers. Although there is some systematic evidence on the receipt of domiciliary care by clients [19] there is little data on patterns of care delivery. As such we make several assumptions based on anecdotal accounts of current practice from practitioners. The assumptions are as follows:

1. clients require/receive between one and four visits per day, these can be fulfilled by the same or different care workers and
2. care workers are either full-time or part-time with their number of visits per day being ten and five, respectively. This approximates the typical eleven-twelve visits per day for full-time and five-six visits per day by part-time care workers. In the former case, these are to seven or eight different households, while in the latter most visits are to different households.

The networks are generated by allocating stubs to each household. This is done by choosing numbers from 1 + Bin(3, *μ*), with some set value for *μ* such that the mean 1 + 3*μ* is around 3. There are care workers of two types: full-time and part-time. These are also allocated stubs (10 for full-time and 5 for part-time care workers). These stubs are then placed in two separate lists (one for households and one for care workers) with each stub being labelled by the node index (e.g. if node *i* is a household with three links the three copies of *i* will be added to the households' stub list). We then choose stubs at random from the household and from the care worker lists, without replacement. This means that duplicate links are possible, and some compatibility conditions need to be observed (i.e. the number of stubs from households has to equal the number of stubs from care workers). Further details are given in the Supplementary material.

**Fig. 2.**
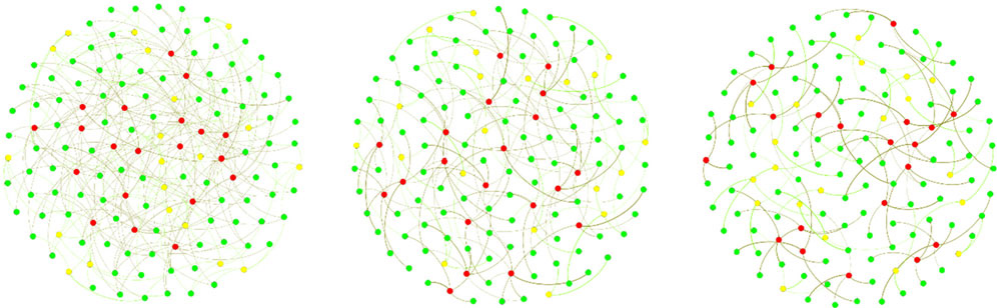
Example of networks with *N*_HH_ = 100 households (green nodes), and equal number of full time (red nodes) and part time (yellow nodes) care workers *N*_FTCW_ = *N*_PTCW_ = 20. *μ* = 2/3 in Bin(3, *μ*) with *p*_overlap_ = 0, 0.5, 1 from left to right.

The network has an extra degree of freedom which allows us to vary the number of repeat visits (later referred to as ‘overlap’). This is done as follows. Once a household and a care worker are connected for the first time, the algorithm searches out all the other stubs from this household and care worker and adds them as extra link with probability *p*_overlap_. High values of *p*_overlap_ corresponding to the case where care workers go back to the same household, as much as possible. Low values of *p*_overlap_ mean visits by care workers are distributed as much as possible and by avoiding repeat visits. Figure 2 shows the clear difference between the no repeat (left panel) and repeat (right panel) scenarios. We note that thicker edges are weighted from one to four, which reflects the maximum number of visits required by a household. The construction implies that the weight of edges in all three networks is the same which means that more overlap between visits leads to sparser networks, with fewer visible links, but with links of higher weight. This simple model allows us to vary the number of repeat visits and thus allows us to investigate its impact on infectious disease outbreaks in the domiciliary care sector.

We are now in a position to impose an epidemic dynamic on the top of the contact structure, and we do this by using an SEIR model where the nodes can be: susceptible (S), exposed (E), infected/infectious (I) and recovered (R). The contact pattern between households and care workers is given by the weighted adjacency matrix *A* = (*a*_*ij*_)_*i*,*j*=1,2, …, *N*_, where *N* is the number of nodes in the network. The transitions between different states for each of the nodes are given in Table 1.

**Table 1.** Transitions at the level of nodes.

Transition	Rate
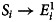	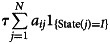
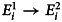	*σK*_1_
⋅⋅ ⋅	⋅⋅ ⋅
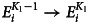	*σK*_1_
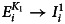	*σK*_1_
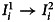	*γK*_2_
⋅⋅ ⋅	⋅⋅ ⋅
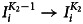	*γK*_2_
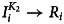	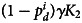
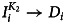	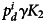

The subscripts *i* = 1, 2, …, *N* are node labels, *τ* is the disease transmission rate per single link in the contact network, 1/*σ* is the incubation period represented by *K*_1_ stages and 1/*γ* is the recovery period represented by *K*_2_ stages. 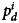 is the probability that an individual dies after leaving the (I) state, while 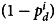 is the probability that they recover. This probability is different for those receiving care and the care workers [13].

This model is simulated numerically, using the Gillespie algorithm on the generated contact networks with different overlap. From a mathematical viewpoint, this model is a continuous time Markov Chain which we simulate using the Gillespie algorithm [20]. Initially all nodes are susceptible except for a randomly chosen one which is set to being infectious. Given a configuration of states over all the nodes in the network, the rate of each possible transition is worked out. For example, a susceptible node will become infected at rate *τ* × (number of infectious neighbours), an exposed node in state *E*^1^ will transition to state *E*^2^ at rate *σK*_1_, and so on. The sum of the rates of all these individual events leads to an overall rate *T* which gives the time to the next event, *δt*, which is chosen from ~*T*exp( − *Tδt*). Next, the update will consist of executing one single event. This is chosen uniformly at random from all possible events but with the probability proportional to the rate of each event. Finally, the rates are updated to reflect the most recent change.

## Results

**Fig. 3.**
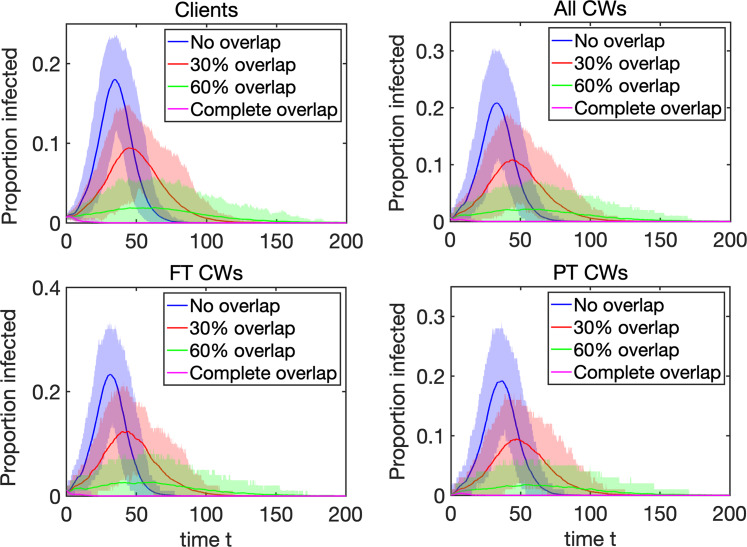
Dynamics of the proportion of infected (exposed E and infectious I) clients, all care workers, as well as full-time and part-time care workers, depending on the level of overlap *p*_overlap_, 90% confidence intervals are also given. An outbreak starts with one infected node chosen at random, and each trajectory represents an average taken over 25 realisations for 10 networks for each value of *p*_overlap_. All epidemics that achieve at least five infected individuals are kept, and time in each individual realisation is re-set to *t* = 0 exactly when the number of infectious nodes is five. Further parameters are: the rate of infection *τ* = 0.2; the rate of recovery from the E state is *σ* = 0.3; the rate of recovery from the I state is *γ* = 0.3; the number of E stages is *K*_1_ = 3 and the number of I stages is *K*_2_ = 5. The probability of dying upon exiting the (I) state is 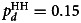 and 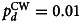 [13] for those receiving care and for care workers, respectively. The underlying networks have *N*_HH_ = 500 households, and equal number of full-time and part-time care workers, *N*_FTCW_ = *N*_PTCW_ = 100, with each making *n*_FTCW_ ≅ 10 and *n*_PTCW_ ≅ 5 visits, respectively.

To model the dynamics of the infectious spread, we have considered networks going from no overlap to high overlap, see Figure 2. In the no overlap (unweighted) network case (see left panel), client needs are met by different care workers, and could potentially involve no return visit by the same care worker. In the high overlap (weighted) network case (see right panel), we assume that if a client is already receiving a visit from a particular care worker and if they require additional visits, then these would most likely be provided by the same care worker. To analyse the effect of possible overlap on epidemic dynamics, we have performed simulations as follows. Varying the probability *p*_overlap_ between 0 and 1 in steps of 0.1, for each value of *p*_overlap_, we generated 10 networks, in which weight distribution account for that particular value of *p*_overlap_, and then on each of those 10 networks, we simulated epidemic dynamics 10 times, thus effectively obtaining 100 simulations for each value of *p*_overlap_. These individual simulations were then averaged, and they provided the results shown in Figure 3, which illustrates how proportions of infected clients, all care workers and separately full-time and part-time care workers, change depending on *p*_overlap_. We observe that increasing the degree of overlap reduces epidemic peaks, which can be explained by the fact the contribution of care workers who make repeat visits to the same household to the overall spread of the infection is limited. Repeat visits to the same household make transmission to these households more likely, but this limits the number of links (i.e. opportunities for wider transmission) available for further or wider spread. Fewer links of higher weight thus limit the reach of the epidemic/outbreak, and the higher is the proportion/weight of links connecting the same pairs of care workers and clients, the higher is the reduction in epidemic peak.

**Fig. 4.**
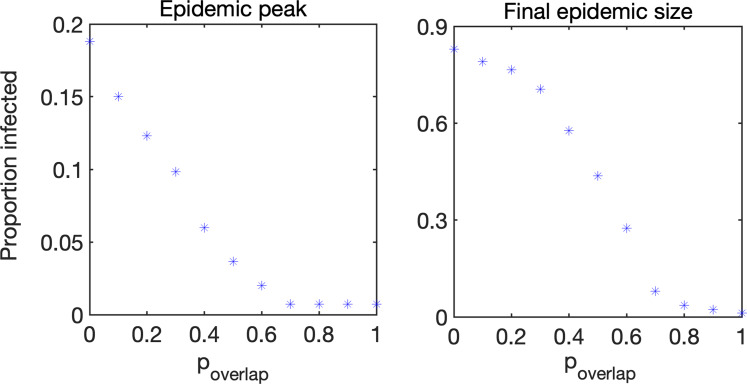
Peak total proportion of infected individuals and final epidemic size defined as those that have entered the infected class. Mortality is shown in Figure 5. Parameter values are the same as in Figure 3.

Figure 4 illustrates this in more detail by showing how the peak in the total proportion of infected individuals, as well as the final epidemic size (overall proportion of initial population who have been infected during the epidemic) are both monotonically decreasing with *p*_overlap_, once again indicating that increasing the degree of overlap between visit allocations reduces the potential for disease spread. The networks that we use have the same number of visits, i.e. the sum of weights on all networks are the same, but the number of links is smaller in the weighted networks, since links account for multiple visits. Also, in weighted networks we varied the proportion of overlap between visits of care workers to the same clients to explore the effect this may have on the dynamics.

**Fig. 5.**
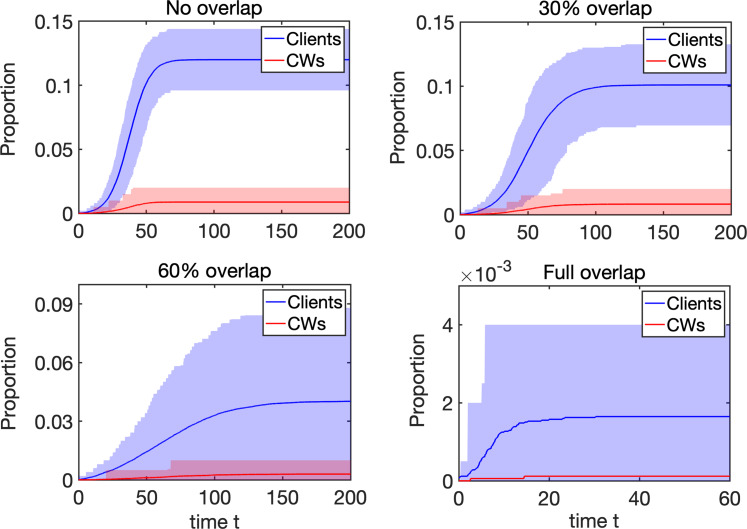
Evolution of the proportion of deaths. Note that deaths are plotted as proportions of all households and all care workers, respectively. The proportions of deaths out of the whole population are 0.08823, 0.07444, 0.02971 and 0.00121 for 0%, 30%, 60% and 100% overlap, respectively. Parameter values are the same as in Figure 3.

Finally, in Figure 5 we report the proportion of deaths for different values of overlap between the visits made by care workers. As explained previously, increasing the number of return visits to the same clients decreases the severity of the outbreak. This is even clearer in Figure 5 where the proportion of deaths decreases with increasing overlap between visits. In fact, the 30%, 60% and 100% overlap leads to a reduction in the average proportion of deaths of ~15%, ~66% and ~98%, respectively, when compared to the case of no overlap at all.

## Discussion

In this paper, we were able to develop and parameterise a simple model of domiciliary-care-related infectious disease transmission, with COVID-19 transmission as a test case. The model shows that maximising the number of return visits (i.e. a care worker visiting the same client during the day or multiple days), while fulfilling care needs, has the potential to limit the peak size and the overall burden of a domiciliary care outbreak. This result supports a policy of maximising the ratio of repeat to one-off visits in designing rosters.

There is a strong consensus that care is of higher quality when provided by a small number of familiar and known individuals [21]. However, the extent to which this is possible will be limited by high vacancy rates (8.2%) and the need to ensure adequate remuneration to retain a workforce of which 58% of domiciliary carers are on zero hour contracts and overall, 58% of care workers in the independent sector in 2019–2020 were paid less than the current national living wage (£8.91) [3]. Other limiting factors may include the need of workers to isolate and lose earnings. Payment of staff for this non-working time will increase employer costs, while if it is unpaid workers will be disincentivised by being forced to bear the costs of reducing infection risk to clients.

This simple model does not consider the interaction between the community and household risks of domiciliary care workers. Future models could usefully include this information, which may be important in understanding the force of infection on domiciliary care client households. Lower-waged workers typically live in more densely crowded accommodation and have experienced higher burdens of COVID-19 infection as low socio-economic conditions and overcrowded housing are risk factor [22, 23]. By contrast domiciliary care clients generally live in small households with few inter-household bridges. We have also not considered inter-worker networks, whether household, social or transport related. These too may be important, as domiciliary care workers mostly provide their own transport, which may be shared with families and co-workers. Work transmission has been shown to be an important contributor to non-household transmission [1].

Although there is increasing data available in relation to COVID-19, often at the granularity of local authority, age, risk groups, etc., it remains a challenge to find data for specific settings, such as domiciliary care. Information about the shift patterns, length of visits, as well as characteristics of those receiving care would be needed, as such information would be key to parameterise a more realistic model. Coupled with longitudinal data, this would make it possible to develop a truly data-driven model which would allow us to fit to realistic observed data, simulate different control scenarios or to implement temporal differences in the model as the epidemic progresses.

This study addresses the transmission risk from care workers to clients in the pre-vaccination era of COVID-19 or a similarly transmissible disease. The rollout of vaccination to care workers, clients and their household contacts can be expected to reduce risk of severe outcomes, not modelled here. However, the impact of vaccination on duration of infectivity, force of infection and attack rates continues to evolve and parameters in the case of COVID-19 will need to follow the emerging epidemiological literature [24–27].

This simple proof of concept model of domiciliary care provides support from an infection control perspective for the current policy consensus that care should be provided by a limited number of familiar carers. The practical implications of doing so in the context of a setting under considerable staffing and financial pressures need to be carefully considered in the development of any new social care policy. There is a need to extend this model to include the social, household and wider community connections of domiciliary care workers and client households. It should also be extended to address the implications of isolation and contact tracing policies for carers, clients, households and for the sustainability of provision in the face of infection risks. Better data, analysis of patterns of use and delivery of home care services is also a further research need. This will enable policy makers, commissioners and clients to understand the most effective actions and workable trade-offs in relation to risk of COVID-19 and other infections that can be facilitated through domiciliary care, affecting clients, carers and their households. This relatively neglected setting deserves focused research, enabling clients, carers and their families to be optimally protected.

## Data Availability

The data that support the findings are available by direct communication with Professor Istvan Zoltán Kiss at I.Z.Kiss@sussex.ac.uk in line with the University of Sussex policy.

## Supplementary material

For supplementary material accompanying this paper visit https://doi.org/10.1017/S0950268821002727. click here to view supplementary material
