# Supplementary material for: How can risk of COVID-19 transmission be minimised in domiciliary care for older people: development, parameterisation and initial results of a simple mathematical model
Source: Epidemiol Infect. 2021 Dec 17;150:e13. doi: 10.1017/S0950268821002727 (PMC8755531; doi:10.1017/S0950268821002727)
Supplement: Supplementary file 1 [file S0950268821002727sup001.docx]

**Supplementary material**

Here we provide some further details for the network construction algorithm.

- Number of households receiving care, $N_{HH}$;
- Number of full-time ($N_{FTCW}$) and part-time ($N_{PTCW}$), giving ${N=N_{HH}+N_{FTCW}+N}_{PTCW}$ nodes in the network;
- Distribution of the number of visits a household or client receives/needs $=1=Bin(3,\mu=2/3)$, which then translates to numbers drawn from this distribution, i.e. $N_{{visits}_{HH}}^{i}$, where ${i=1,2, \ldots, N}_{HH}$;
- Number of visits by full-time care workers is $n_{FTCW}$ and it is $n_{PTCW}$ for part-time care workers;
- We now allocate a proportion $p_{FTCW}=n_{FTCW}N_{FTCW}/(n_{FTCW}N_{FTCW}+n_{PTCW}N_{PTCW})$ of all household stubs to full-time care workers and the remaining ones to part-time care workers. At this point the stubs are not yet allocated to care workers.
- The number of visits made by care workers is now allocated based on $N_{{visits}_{FTCW}}=floor\left( \left( \sum_{i=1}^{N} N_{{visits}_{HH}}^{i} \right)\times p_{FTCW} \right)+remaining-visits_{allocated}-at-random$, with a similar formula for the part-time care workers. This means that some care workers will have $\pm1$ or so stubs compared to $n_{FTCW}$ or $n_{PTCW}$. But such differences are minimised by a careful choice of the number of care workers of different type;
- Create a list/array by placing copies/labels of HH in a list as many times as their number of visits requires;
- Do the same for full-time and part-time CW in a different list;
- Pick elements at random from both lists and connect them up;
- This will produce a network where most links have only been realised once, with some duplicate links occurring;
- This is then refined to allow multiple links between the same household and CW leading to weighted edges. This is done as follows. Once a HH and a CW is connected, the algorithm looks for any of stubs belonging to the same HH and CW and connects them with probability $p_{overlap}$.
